# Therapeutic radiation for childhood cancer drives structural aberrations of NF2 in meningiomas

**DOI:** 10.1038/s41467-017-00174-7

**Published:** 2017-08-04

**Authors:** Sameer Agnihotri, Suganth Suppiah, Peter D. Tonge, Shahrzad Jalali, Arnavaz Danesh, Jeffery P. Bruce, Yasin Mamatjan, George Klironomos, Lior Gonen, Karolyn Au, Sheila Mansouri, Sharin Karimi, Felix Sahm, Andreas von Deimling, Michael D. Taylor, Normand J. Laperriere, Trevor J. Pugh, Kenneth D. Aldape, Gelareh Zadeh

**Affiliations:** 1 MacFeeters Hamilton Centre for Neuro-Oncology Research, Princess Margaret Cancer Centre, University Health Network, Toronto, ON Canada M5G 1L7; 2 Department of Surgery, Division of Neurosurgery, University of Toronto, Toronto, ON Canada M5S 1A8; 3 Princess Margaret Cancer Centre, Toronto, ON Canada M5G 2M9; 4 Department of Laboratory Medicine and Pathobiology, University of Toronto, Toronto, ON Canada M5S 1A8; 5 Department of Neuropathology, Institute of PathologyUniversity Hospital Heidelberg, Heidelberg, 69120 Germany; 6 Clinical Cooperation Unit Neuropathology, German Consortium for Translational Cancer Research (DKTK) German Cancer Research Center (DKFZ), Heidelberg, 69120 Germany; 7 Developmental & Stem Cell Biology Program, Arthur and Sonia Labatt Brain Tumour Research Centre, The Hospital for Sick Children, Toronto, ON Canada M5G 1L7

## Abstract

Cranial radiotherapy improves survival of the most common childhood cancers, including brain tumors and leukemia. Unfortunately, long-term survivors are faced with consequences of secondary neoplasia, including radiation-induced meningiomas (RIMs). We characterized 31 RIMs with exome/*NF2* intronic sequencing, RNA sequencing and methylation profiling, and found *NF2* gene rearrangements in 12/31 of RIMs, an observation previously unreported in sporadic meningioma (SM). Additionally, known recurrent mutations characteristic of SM, including *AKT1*, *KLF4*, *TRAF7* and *SMO*, were not observed in RIMs. Combined losses of chromosomes 1p and 22q were common in RIMs (16/18 cases) and overall, chromosomal aberrations were more complex than that observed in SM. Patterns of DNA methylation profiling supported similar cell of origin between RIMs and SMs. The findings indicate that the mutational landscape of RIMs is distinct from SMs, and have significant therapeutic implications for survivors of childhood cranial radiation and the elucidation of the molecular pathogenesis of meningiomas.

## Abstract (web summary)

Radiation-induced meningiomas are often more aggressive than sporadic ones. In this study, the authors perform an exome, methylation and RNA-seq analysis of 31 cases of radiation-induced meningioma and show *NF2* rearrangement, an observation previously unreported in the sporadic tumors.

## Introduction

Radiotherapy improves survival of the most common childhood cancers, including brain tumors and leukemia. Unfortunately, long-term survivors are faced with consequences of secondary neoplasia, including radiation-induced meningiomas (RIMs). RIMs are the most common brain neoplasm caused by ionizing radiation (al-Mefty et al^1^. 1990; Lee et al.^2^ 2004). These tumors occur 10–30 years following radiotherapy for primary childhood cancer^3–5^, and demonstrate distinct and more aggressive clinical features than sporadic meningiomas^6–14^. Furthermore, RIMs display more aggressive clinicaland biologic behavior than sporadic meningiomas, including higher recurrence rates, greater cellularity, more pleomorphic nuclei and frequent mitoses, and are more often atypical and multifocal^6–14^. NF2 mutations have been reported to be absent or significantly lower in RIMs (25%) compared to sporadic meningiomas^15^, supporting that fact that the genomic landscape of RIMs is possibly unique from sporadic meningiomas. While the genome of sporadic meningiomas has been well characterized with genetic alterations defining distinct molecular subgroups, the genomic landscape of RIM remains poorly characterized^16^. To date, the most common alterations in sporadic meningiomas are NF2 mutations or loss of 22q, with non-NF2 mutant meningiomas harboring mutations in in AKT1, TRAF7, KLF4, POLR2A and SMO^16–18^. Therefore, we performed an integrative and multiplatform approach to investigate the somatic genetic landscape of RIM and its distinctness to sporadic meningioma.

## Results

### Mutational profile of RIMs is distinct from sporadic meningiomas

**Fig. 1:**
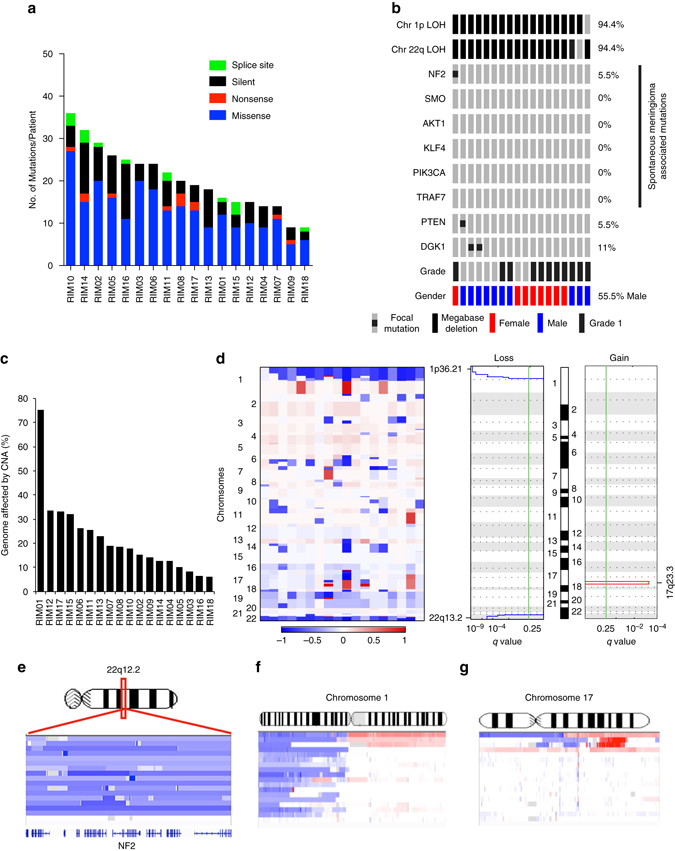
Mutation profile of radiation-induced meningioma. Whole-exome sequencing of RIMs reveals non-synonymous mutations **a** and focal mutations **b**, **c** Percentage of the genome affected by copy number alterations. **d** Copy number alterations within 18 RIMs and statistical significance (*q* value) were calculated by GISTIC 2.0. **e**–**g** High-resolution views of chromosomes harboring recurrent CNAs

We analyzed 31 RIMs, including a discovery cohort of 18 tumors from 16 patients who had received childhood radiation therapy for cancer, the majority (74%) being survivors of leukemia or medulloblastoma (Supplementary Data 1). We also assessed 30 sporadic (non-radiation-associated) meningiomas as a control cohort. Whole-exome sequencing (WES) at a median depth of 112× (range 110–120) showed no significant germline aberrations in genes associated with a cancer predisposition or DNA repair, including *TP53, NF2* and *PTEN*, among others. We identified 265 non-synonymous somatic single nucleotide variants (SNVs) within the cohort of 18 tumor samples, with an average of 14.7 SNVs per tumor (Fig. 1a, Supplementary Data 2). The overall mutation rate of non-synonymous mutations in our cohort was 0.47 per Mb. Most notable was a marked absence of focal gene mutations typically found in spontaneous meningiomas (Fig. 1b), such as *SMO, TRAF7, KLF4, PIK3CA* and *AKT1*. Consistent with prior reports^15^ the rate of NF2 focal mutation was low (1/18 cases). A truncating mutation in *PTEN* was also noteworthy (Supplementary Data 2), a rare event in sporadic meningioma, but previously observed in an RIM^19^.

### Copy number alterations in RIMs

As a possible alternative mechanism to somatic SNVs and small insertion/deletions, we analyzed the exome data for copy number alterations (CNAs). All 18 RIMs exhibited frequent megabase-level CNAs with an average of 22% of the genome affected by CNAs (Fig. 1c), which is >5-fold (*p* < 0.01, Student’s *t*-test) above that observed in the sporadic meningioma genome^17^. When controlling for grade by removing 5 RIMs that were grade II, the remaining 13 grade I RIM patient samples still displayed significantly high rate than the sporadic meningioma genome (4.2×, *p* < 0.01, Student’s *t*-test). The most common CNAs were loss of chromosomes 1p (17/18 RIMs) and 22q (17/18 RIMs) (Fig. 1d–g, Supplementary Data 3). Previous reports of single cases or small series have implicated 1p in RIMs^20–23^, and a cytogenetic analysis of six cases has implicated 1p13 as a region involved in these tumors^21^. Overall, 16/18 cases showed dual loss of chromosomes 1p and 22q, suggesting that co-occurrence of 1p and 22q loss radiation-induced meningioma is a near-universal feature (89%, *p* < 0.05, Fisher’s exact test) of radiation-induced meningioma. Two patients that possessed multiple RIMs in distinct locations, exhibited divergent mutational profiles and CNA patterns between the paired samples, suggesting that multiple RIMs are not seeded by a common tumor cell ancestor, but rather represent distinct, synchronous neoplasms (Supplementary Fig. 1).

**Fig. 2:**
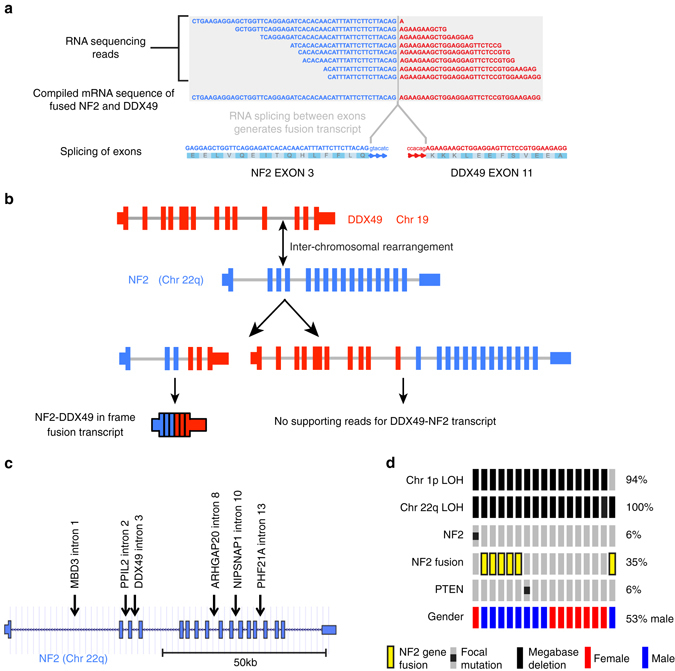
*NF2* gene fusions. **a** RNA sequencing supports the detection of an inter-chromosomal gene fusion between *NF2* and *DDX49*. **b** Schematic representation of hypothetical genomic rearrangement that generates chimeric RNA transcript. **c** Genomic location of *NF2* intronic breakpoint for all 6 detected gene fusions. **d** *NF2* fusion genes are mutually exclusive to *NF2* and *PTEN* focal mutations

Notable from RNA sequencing data were *NF2* gene fusion events, identified in 6 of the 17 RIMs that passed RNA-Seq quality control, as supported by ≥8 spanning RNA-sequenced mate pairs (Supplementary Data 4). In one case, an inter-chromosomal gene fusion generated an in-frame *NF2* (chr22) with the DEAD-box helicase *DDX49* (chr19) transcript (Fig. 2a). The first three *NF2* exons were expressed in the *NF2*-*DDX49* fusion; however, no sequence reads support the reciprocal *DDX49-NF2* transcript, suggesting that this fusion gene is not expressed at an appreciable level. In all six cases of *NF2* fusion a complete *NF2* exon is spliced to a complete exon of a reciprocal gene, indicating that the breakpoints of genomic rearrangement are intronic. The predicted genomic breakpoints of the six *NF2* genomic rearrangements varied between first intron to the thirteenth intron (Fig. 2c). All six RIMs from the discovery set found to harbor an *NF2* rearrangement also possessed monosomy of chromosome 22q (Fig. 2d), which in combination with *NF2* genomic rearrangement is predicted to result in homozygous disruption of *NF2*. Furthermore, when we examined the magnetic resonance imaging scans of patients it was clear that RIMs possessing an *NF2* gene fusion exhibited ill-defined borders (*p* < 0.01, Fisher’s exact test) and a correlation towards an anatomic frontal location in the brain (67%, Supplementary Fig. 2).

### ***NF2*** rearrangements in RIMs

*NF2* RNA sequencing reads confirmed the *NF2 G*>A point mutation as detected by WES within intron 11 splice donor site for one case (RIM1) (Supplementary Fig. 3A). We examined the coverage of RNA sequencing reads mapping to the *NF2* locus to determine whether there was a transcriptional consequence of the splice donor site mutation. High levels of *NF2* sequence reads mapping to the 1320 bp intron 11 revealed that it was incorporated into the *NF2* RNA transcript, indicating the loss of function of intron 11 splice donor site. The inclusion of intron 11 introduces a premature stop codon that disrupts *NF2* function through generation of a truncated protein (Supplementary Figs 3B, C).

**Fig. 3:**
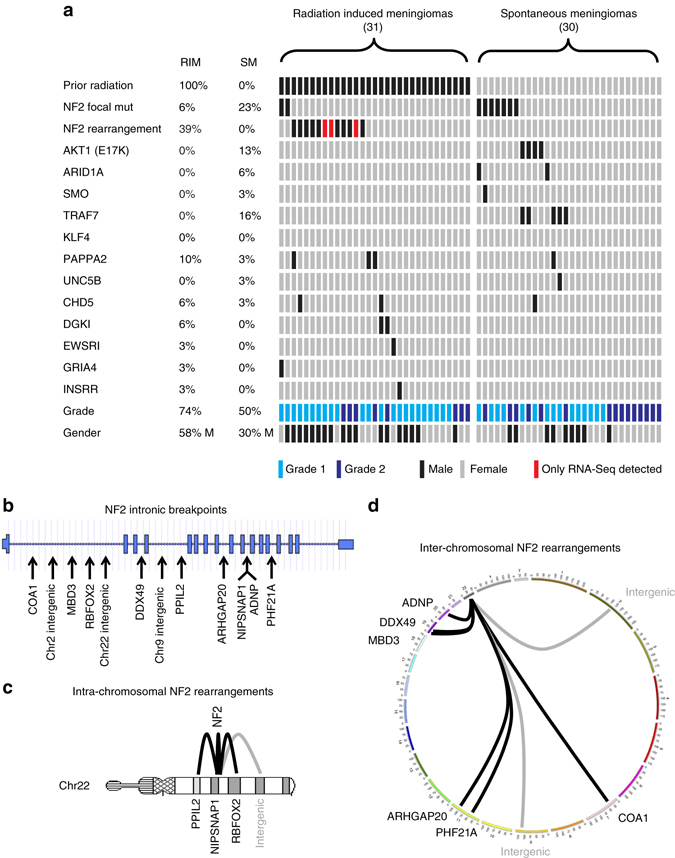
*NF2* structural rearrangements. **a** Mutation profile of radiation-induced meningioma and sporadic meningioma by targeted sequencing. **b** Schematic representation of NF2 intronic breakpoints. **c**, **d** Schematics represent *NF2* genomic rearrangements with reciprocal loci

To discover additional *NF2* intronic rearrangements in an expanded cohort of meningiomas we designed a targeted sequencing panel to capture both exons and introns of *NF2* (Supplementary Data 5) within 31 RIMs and 30 sporadic meningiomas. Supporting our WES data (Fig. 1 core radiation-induced meningioma cohort), we found the mutation rate of NF2 was 3.5 times lower in RIMs, with only two mutations (focal *NF2* mutations) identified in RIMs, but confirmed their common occurrence (7/30, 23%) in sporadic meningiomas (Fig. 3a, Supplementary Data 6). The constitutively activating *AKT1* E17K mutation was observed in 13% of sporadic meningiomas (4/30), but was absent in our cohost of radiation-induced tumors. Other mutations associated with meningiomas (*ARID1A, SMO* and *TRAF7*) were also restricted to sporadic meningiomas, suggesting that radiation therapy induces tumorigenesis through a different set of genetic alterations to those of sporadic meningioma. Overall, we observed intronic rearrangements of *NF2* in 12/31 RIMs, in contrast to 0/30 in sporadic tumors (Fig. 3a, Supplementary Data 6–8, *p* < 0.001, Fisher’s exact test). Of the 12 fusions only one (NF2-DDX49) was in frame Supplementary Data 9). Moreover, all NF2 fusion partners on 22q, had the accompanying wildtype gene deleted through 22q loss and for the non-22q fusion partners only one (NF2-DDX49) was unbalanced as the remaining wildtype copy of DDX49 was deleted (Supplementary Data 9).

### The distinctions of the methylome in RIMs

To further characterize these tumors, we interrogated the methylome of our cohort and compared these to a set of sporadic tumors. Hierarchical clustering demonstrated similarity in DNA methylation profiles between radiation-induced vs. sporadic tumors, suggesting a common cell of origin (Supplementary Fig. 4A). Overall, clustering demonstrated two robust methylation subtypes of radiation-induced tumors, with methylation subgroup one enriched for NF2-disrupted tumors (NF2 fusion and focal mutations) (Supplementary Figs 4,5). Distinguishing groups one and 2 were 600 differentially methylated probes (Wilcoxon rank sum test, 30% methylation change, *p* < 0.05 with adjusted Bonferroni correction) whose associated genomic loci are enriched for epigenetic biological pathways (DNA methylation and histone modification) (Supplementary Figs 6A, B). When methylation sub-groups 1 and 2 were examined at the transcriptome level we identified 249 differential expressed transcripts between the two-methylation subgroups (DESeq, *p* < 0.05, adjusted Bonferroni Correction, Supplemental Fig. 6C) with enrichment in biological pathways similar to our methylation analysis including epigenetic alterations (Supplementary Fig. 6D).

## Discussion

In a previous study comprised predominately of sporadic meningiomas, an *NF2* rearrangement was previously observed in a single case, interestingly from a patient with a prior history of cranial radiation^17^. Consistent with our findings, it occurred in a male patient, with an *NF2* rearrangement in intron 1, the largest *NF2* intron that harbored 5/12 of the *NF2* structural rearrangements in our cohort. This example further confirms the association of *NF2* intronic rearrangements to patients who received prior radiation therapy.

Our study demonstrates that RIMs possess a mutational signature that is distinct from sporadic meningioma, displaying *NF2* inactivation through structural rearrangements. *NF2* intronic rearrangements are promiscuous, exhibiting fusion with intra- and inter-chromosomal regions. Interestingly, only one fusion was in frame, NF2-DDX49, and although not recurrent we hypothesize this fusion may harbor some oncogenic properties as seen with other in frame fusions in other central nervous system and peripheral nervous system cancers^24–27^. Loss of 1p and 22q was highly frequent, and notable is an absence of mutations in additional genes common to sporadic meningioma. We hypothesize that radiation therapy triggers genomic structural rearrangements through error prone repair of double strand DNA breaks. This is supported by our finding that CNAs are elevated in radiation-induced tumors, in comparison to sporadic meningioma. Radiation may generate genomic changes involving *NF2* disruption and other changes that predispose to meningioma development over several decades. In a recent study, mutational signatures of ionizing radiation in second malignancies demonstrated that ionizing radiation generates distinctive mutational signatures that explain its carcinogenic potential^28, 29^. Our cohort was lacking whole-genome profiling of these tumors, which is the best way to detect these mutational signatures. Although highly likely our RIM cohort would display a radiation mutational signature, we were unable to detect it as WES only covers approximately 1% of the genome and we had a very low mutation rate in the coding exons. Intriguingly, 10 of the 12 NF2-rearranged RIMs were in males (*p* < 0.05). Although sporadic meningiomas are more common in women while the female-male ratio of RIMs is balanced. Therefore it has been suggested that men have excess relative risk of developing meningioma after radiation^30^.

Since druggable targets found in sporadic meningioma, such as *AKT1* and *SMO*, were not observed within our cohort of 31 RIMs, our findings are relevant in light of current and future targeted clinical trials for meningioma, and indicate that effective therapies for meningioma in childhood cancer survivors may be distinct from precision medicine approaches employed for sporadic meningioma. Future studies are necessary to correlate the dose, latency and dosimetry of radiation with frequency of *NF2* rearrangements and increased CNA load. We note that one of our NF2-rearrranged cases was in association with prior low-dose radiation for a certain condition (tinea capitis), but larger studies and comparisons will be required to fully elucidate this. Overall, applying these results towards a therapeutic strategy that allows early intervention in childhood survivors of cancer will be the most effective means to understand and assess the risk of meningioma following radiation in these patients.

## Methods

### Sample selection and preparation

This study was reviewed and approved by the University Health Network research ethics board. Specimens were obtained from patients, with written informed consent. A primary cohort of 18 radiation-induced meningioma tumors was selected from a clinically annotated database of over 1200 meningioma cases. Criteria for a meningioma to be classified as radiation-induced included evidence that patient received cranial irradiation at least 2 years before initial meningioma diagnosis. Tumor samples were only considered for this study if a detailed clinical history was available. Meningioma samples were classified and graded according to World Health Organization (WHO) criteria. The clinical and histopathological data pertaining to all analyzed meningiomas is outlined in Supplementary Data 1.

Representative fresh-frozen tissue with an estimated purity of ≥90% was selected from each case for molecular profiling. Genomic DNA and total RNA were extracted from 4 mm^3^ tissue samples with standard molecular techniques using qSYNC DNA extraction kit (Geneaid) and RNeast kit (Qiagen) respectively.

### Whole-exome sequencing

Whole-exome sequencing of 18 RIMs and matched blood samples was performed by EA Quintiles Genomic services, Durham, NC. Qualimap and Picard confirmed that sequencing results were of high quality and clean of contaminants. Based on read quality, duplication rate and target coverage, normal and tumor samples are indistinguishable. Tumor/Normal exome-seq BAMs were processed by Mutect v1.1.41 to identify somatic point mutations. In order to identify copy number alterations, aligned exome-seq BAM files for tumor/normal pairs were processed by samtools mpileup v0.1.18 2 the output of which was input into Varscan2 somatic v2.3.6 3 which generates depth-ratio. Somatic/germ-line single nucleotide polymorphism (SNP) calls were processed using the R package Sequenza v2.1.0 4 in order to generate segmented copy-number profiles and depth ration plots for each tumor sample. Segmented copy number data were then input into GISTIC v2.0.22 5 in order to identify genomic regions which exhibited significant copy number alterations across the cohort.

### Methylation profiling

Genomic tumor DNA (0.5 μg) was bisulphite converted (Qiagen, EpiTect plus) and hybridized to the Infinium HumanMethylation450 BeadChip (Illumina). Array data can be accessed at the Gene Expression Omnibus repository (GSE83933). Arrays were normalized using the open source statistical programming language R. Raw IDAT data files were processed using the minfi Bioconductor package and SWAN normalized. Methylation values were then exported as β-values (CpG methylation levels). Methylation probes that overlapped single nucleotide polymorphisms or mapped to the sex chromosomes (X and Y) were removed before further analysis. Consensus clustering, non-negative matrix factorization and principal component analysis for subgroup identification was performed in GenePattern and MultiExperimentViewer. SigClust (version 1.1.0) was used to compute the significance of the identified clusters.

### Targeted sequencing

The targeted sequencing cohort of meningiomas comprised of 31 RIMs (including the 18 primary cohort) and 30 spontaneous meningioma. Clinical and histopathological data pertaining to this cohort of 61 meningioma can be found in Supplementary Data 8. Targeted sequencing procedure was performed as described in Agnohtri et al.^31^. A custom meningioma xGen Lockdown Panel of DNA-biotinylated capture probes was generated from Integrated DNA Technologies (IDT, Coralville, Iowa) to enrich for all exons of the following genes: NF2, AKT1, ATM, CHD5, DGKI, EWSR1, GRIA4, INSRR, KLF4, PAPPA2, RAD54L, SMO, TRAF7 and UNC5B). In addition, to enable the identification of NF2 intronic rearrangements we designed capture probes to span the introns of NF2 (Supplemental Data 6). Targeted sequencing was performed at the Princess Margaret Genomics Centre (PMGC, www.pmgenomics.ca), Toronto Canada on an Illumina HiSeq2500.

### RNA sequencing

RNA sequencing of 18 RIMs was performed by EA Quintiles Genomic services, Durham, NC. Libraries were generated from total RNA and constructed using the manufacturer’s protocols. Sequencing was done on the Illumina HiSeq platform. One RIM sample failed to pass the sequencing quality control, with the remaining 17 RIMs passing. RNA-Seq fastq files were processed using tophat2 v2.0.8b 6 in fusion-search mode in order to identify putative gene fusions.

### Data availability

All data has been deposited in publically accessible databases. Methylation data are accessible through the Gene Expression Omnibus (accession GSE83933). Exome and RNA Sequencing data is accessible through the European Genome-phenome Archive (EGAS00001002317 and EGAS00001002318, respectively).

## Electronic Supplementary Material

Supplementary Information

Supplementary Data 1

Supplementary Data 2

Supplementary Data 3

Supplementary Data 4

Supplementary Data 5

Supplementary Data 6

Supplementary Data 7

Supplementary Data 8

Supplementary Data 9
