# Supplementary material for: Therapeutic radiation for childhood cancer drives structural aberrations of NF2 in meningiomas
Source: Nat Commun. 2017 Aug 4;8:186. doi: 10.1038/s41467-017-00174-7 (PMC5543118; doi:10.1038/s41467-017-00174-7)
Supplement: Supplementary file 1 — Supplementary Information [file 41467_2017_174_MOESM1_ESM.pdf]

File Name: Supplementary Information
Description: Supplementary Figures

File Name: Supplementary Data 1
Description: Patient clinical data for the primary radiation-induced meningioma cohort.

File Name: Supplementary Data 2
Description: Mutect mutation calls from whole exome sequencing.

File Name: Supplementary Data 3
Description: GISTIC 2.0 significant copy number alterations detected by WES.

File Name: Supplementary Data 4
Description: Tophat2 identified gene fusion candidates.

File Name: Supplementary Data 5
Description: IDT capture probe panel for targeted sequencing.

File Name: Supplementary Data 6
Description: Mutect mutation calls from targeted sequencing of RIMs and sporadic meningiomas.

File Name: Supplementary Data 7
Description: Targeted sequencing reads supporting NF2 rearrangements.

File Name: Supplementary Data 8
Description: Clinical data and mutation profile of meningioma targeted sequencing.

File Name: Supplementary Data 9
Description: Summary of NF2 fusions/breakpoint and copy number alterations.

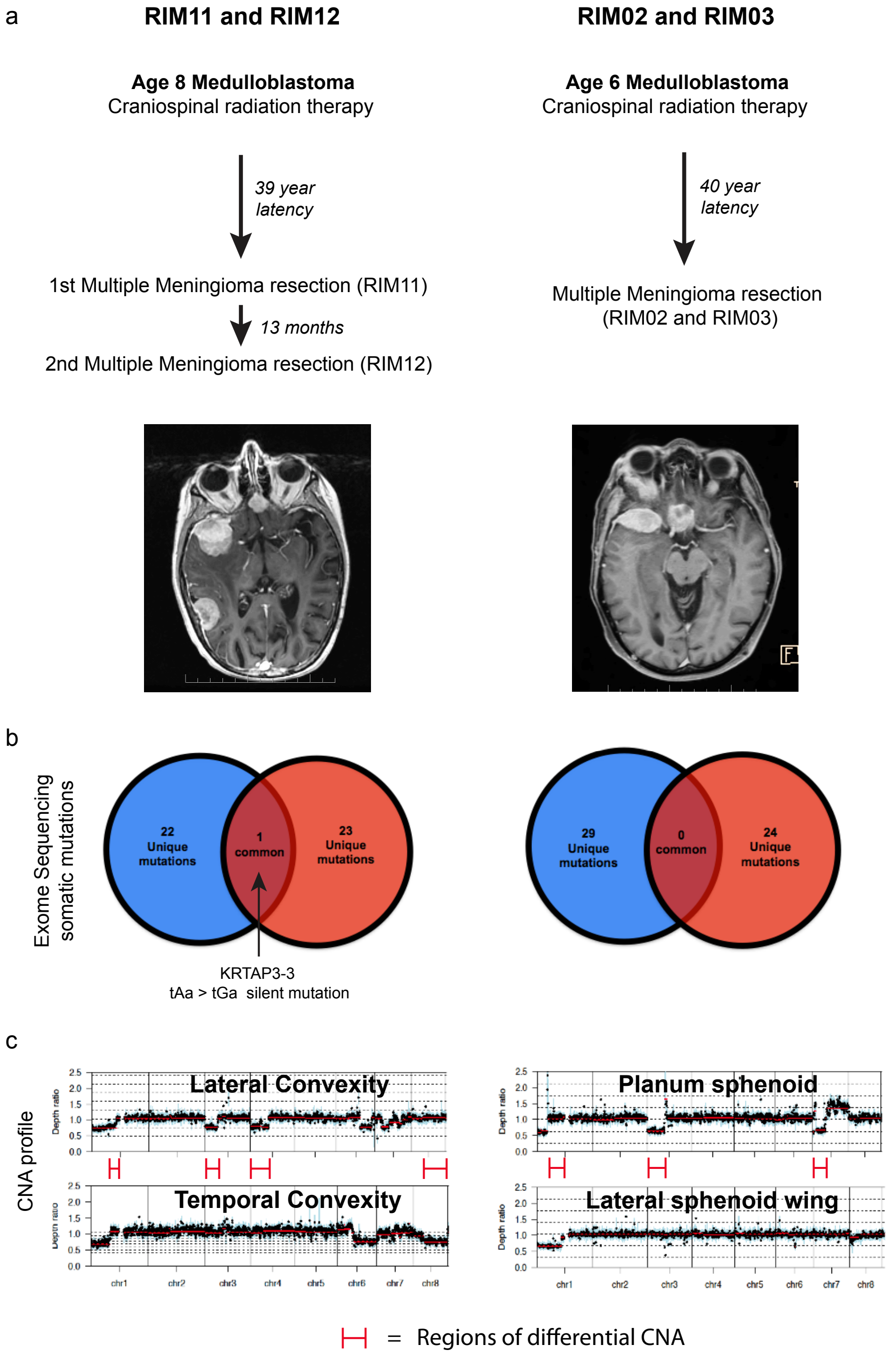

Supplementary Figure 1

Characterization of multiple radiation-induced meningioma cases from single patients.

(a). Clinical timeline of radiation therapy to meningioma resection. **(b)** Somatic focal mutations detected by exome sequencing **(c)** CNA profiles as determined by exome sequencing.

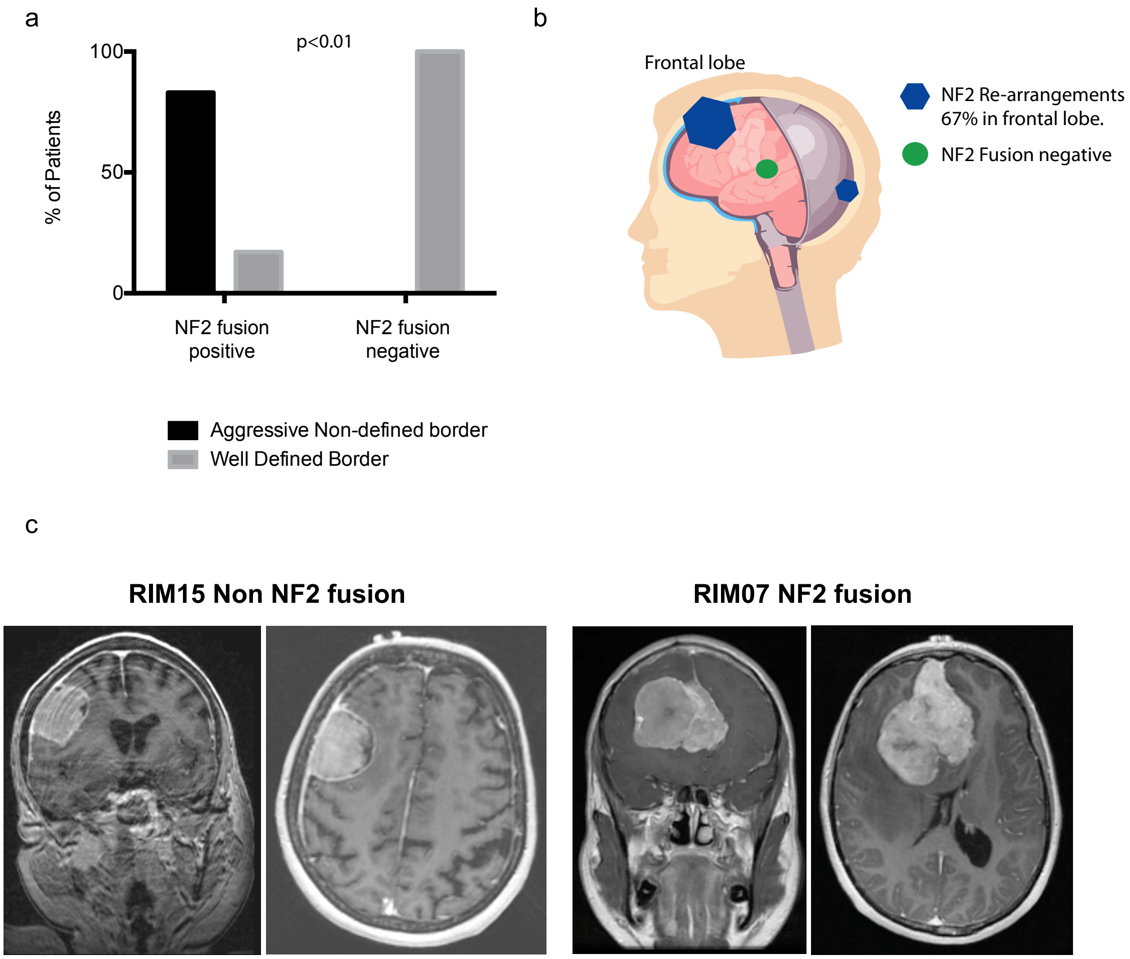

Supplementary Figure 2

Correlation of NF2 gene fusion with tumor characteristics.

(a) Tumor border definition with respect to NF2 fusion event. **(b)** Schematic representing tumor anatomical position. **(c)** Representative MRIs of RIMs.

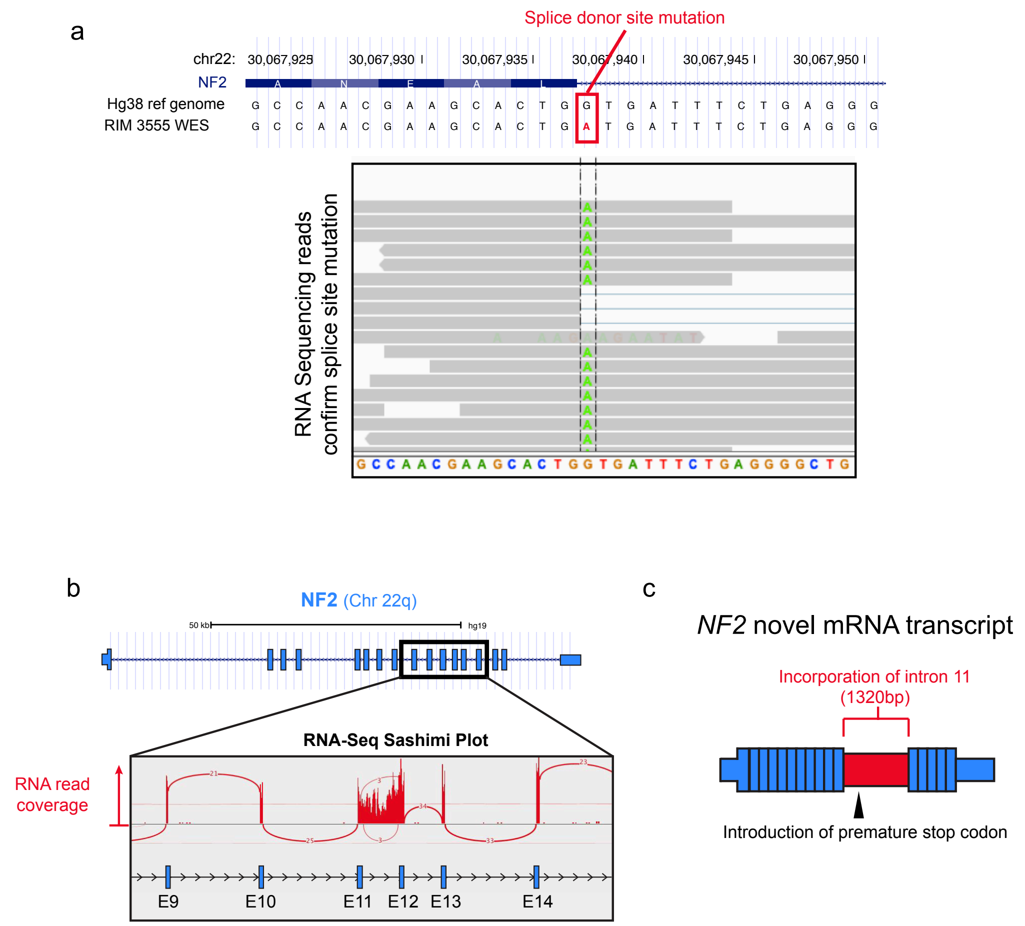

Supplementary Figure 3

Transcriptional consequence of intronic NF2 mutation.

(a) RNA sequencing reads confirm the NF2 splice donor site mutation (G>A) detected by WES. **(b)** RNA Sequencing reads reveal the failure to splice out intron 11 from the mRNA transcript. **(c)** Schematic representation of NF2 RNA transcript with inclusion of intron 11.

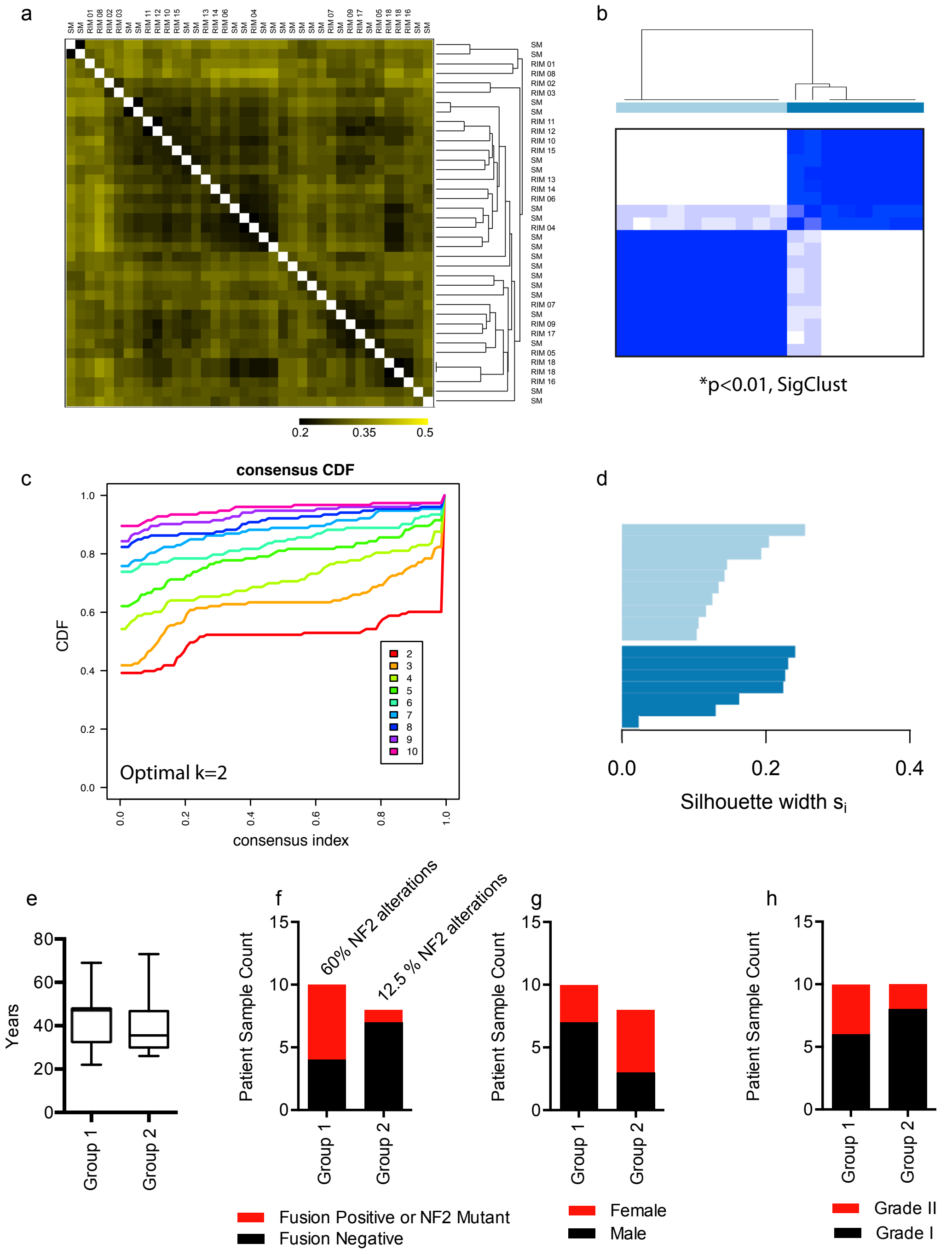

Supplementary Figure 4

DNA Methylation profiling of Radiation Induced Meningioma.

(a) Sample distance matrix with hierarchical clustering (Pearson correlation). **(b)** Heatmap of Consensus K-means clustering representing two subgroups. *p<0.05, SigClust results. **(c)** Consensus CDF plot supporting two groups are the most stable as identified through PAC clustering. **(d)** Silhouette plot of how each sample is representative of their group assignment. Negative values indicate poor group assignment. **(e)-(h)** Association of methylation sub-groups with clinical characteristics of RIM.

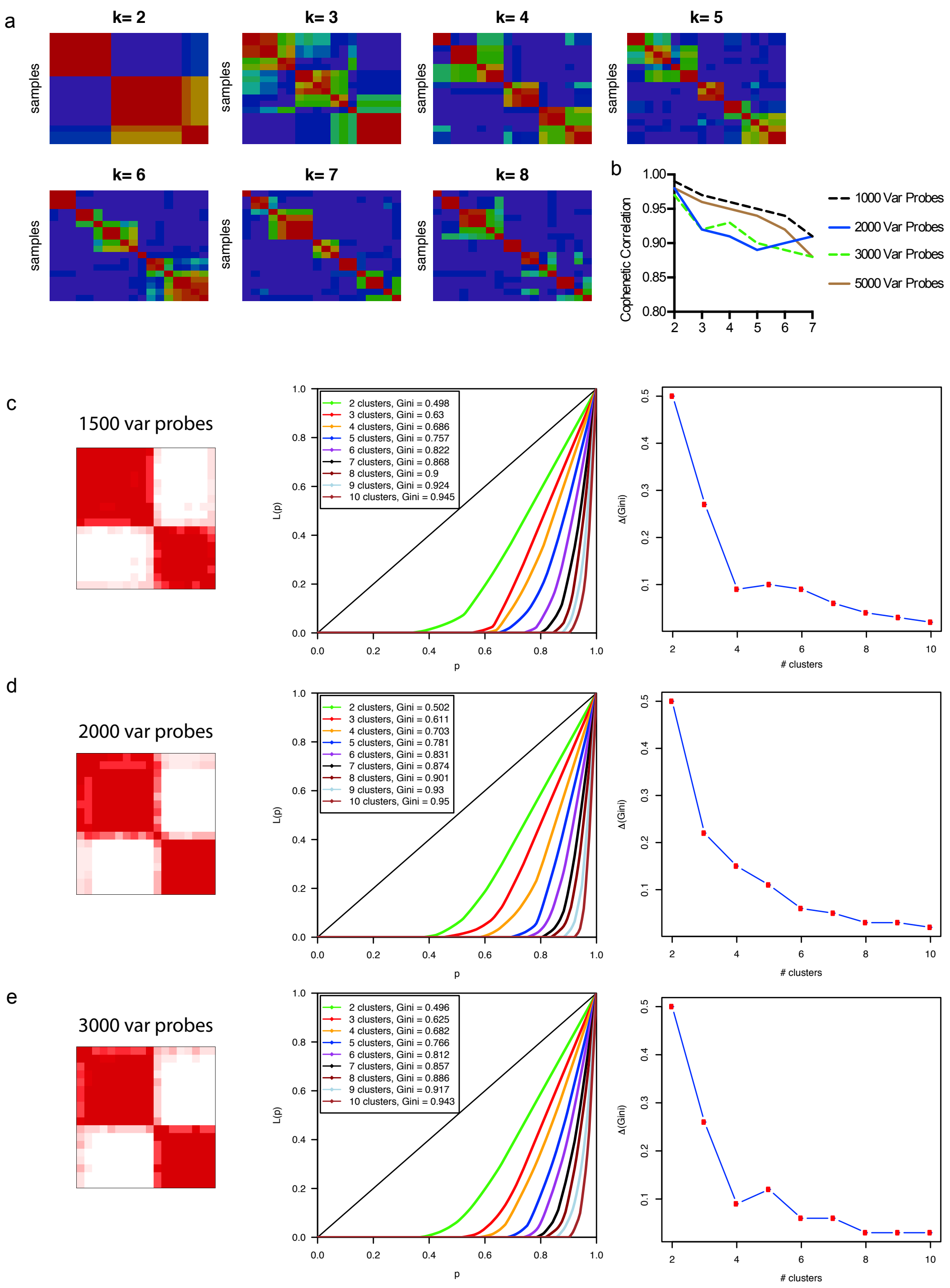

Supplementary Figure 5

Consensus clustering of methylation profiles.

(a) Nonnegative Matrix Factorization (NMF) for 1500 most variable probes supports do groups of RIM. **(b)** Varying methylated probes still supports two groups as depicted by the co-phenetic correlation plot. **(c)** Consensus hierarchical clustering of 1500 most variable methylated probes supports two groups as depicted by the heatmap (left), Lorenz curve (middle) and Change in Gini plot (right). **(d)** Consensus hierarchical clustering of 2000 most variable methylated probes supports two groups as depicted by the heatmap (left), Lorenz curve (middle) and Change in Gini plot (right). **(e)** Consensus hierarchical clustering of 3000 most variable methylated probes supports two groups as depicted by the heatmap (left), Lorenz curve (middle) and Change in Gini plot (right).

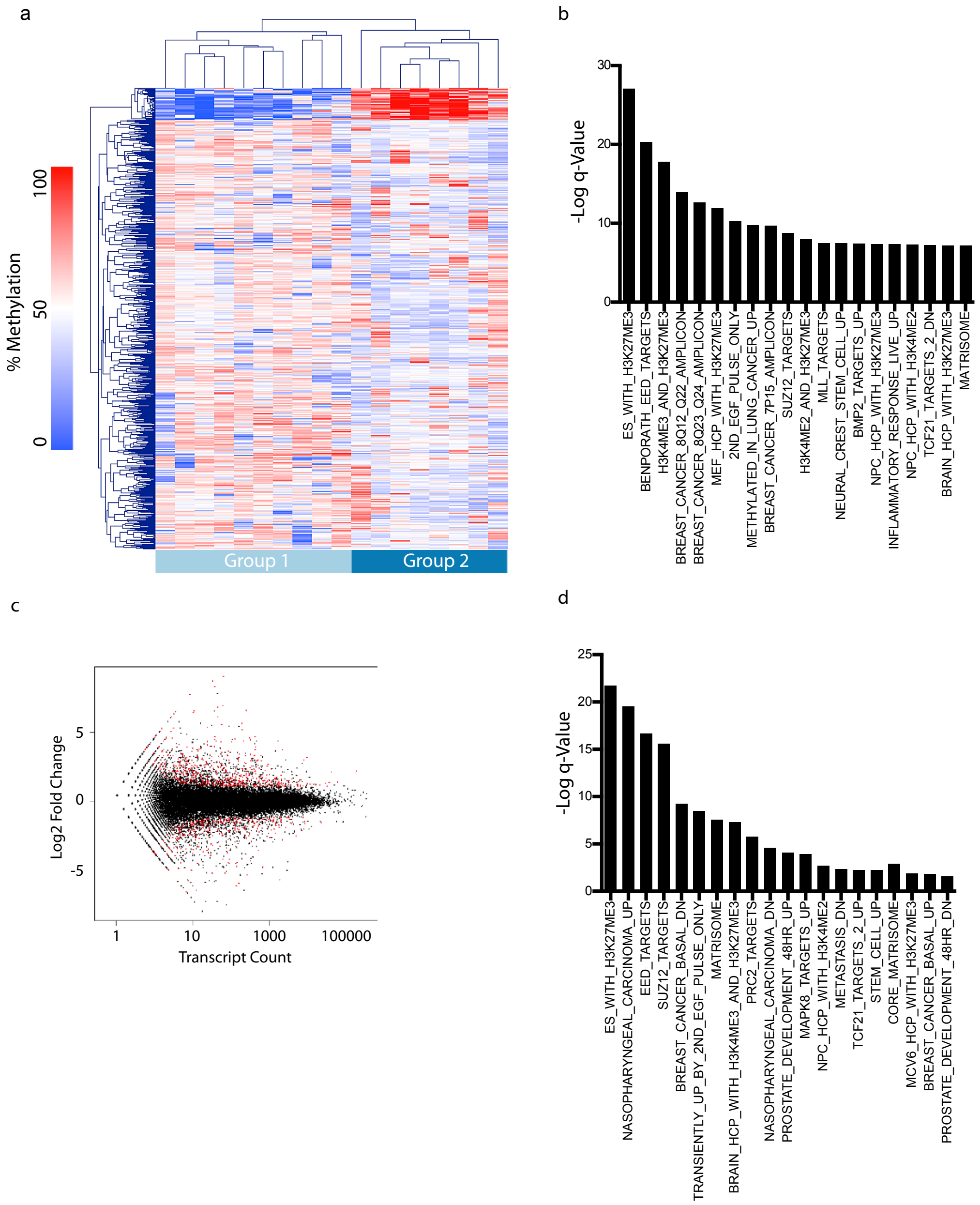

Supplementary Figure 6

Pathway analysis of RIM methylation sub-groups 1 and 2

(a) Hierarchical clustering of significant differentially methylated probes between radiation-induced meningioma methylation groups 1 and 2. Significance was established as: minimum 30% methylation change with an adjusted Bonferroni correct p-value of $p<0.05$. **(b)** GSEA of genes identified in panel a. **(c)** DESeq plot identifying differentially expressed transcripts between radiation-induced meningioma methylation groups 1 and 2. Significance was established as: minimum 2 fold change at the transcript level with an adjusted Bonferroni correct p-value of $p<0.05$. **(d)** GSEA of genes identified in panel **c**.
